# Synthesis of Chitosan-Based Gold Nanoparticles: Antimicrobial and Wound-Healing Activities

**DOI:** 10.3390/polym14112293

**Published:** 2022-06-05

**Authors:** Amr H. Hashem, Amr M. Shehabeldine, Omar M. Ali, Salem S. Salem

**Affiliations:** 1 Botany and Microbiology Department, Faculty of Science, Al-Azhar University, Nasr City, Cairo 11884, Egypt; 2 Department of Chemistry, Turabah University College, Turabah Branch, Taif University, P.O. Box 11099, Taif 21944, Saudi Arabia; om.ali@tu.edu.sa

**Keywords:** nanocomposite, chitosan, antibacterial activity, antifungal activity, wound healing, cytotoxicity

## Abstract

The global spread of multidrug-resistant bacteria has become a significant hazard to public health, and more effective antibacterial agents are required. Therefore, this study describes the preparation, characterization, and evaluation of gold nanoparticles modified with chitosan (Chi/AuNPs) as a reducing and stabilizing agent with efficient antimicrobial effects. In recent years, the development of an efficient and ecofriendly method for synthesizing metal nanoparticles has attracted a lot of interest in the field of nanotechnology. Colloidal gold nanoparticles (AuNPs) were prepared by the chemical reduction of gold ions in the presence of chitosan (Chi), giving Chi/AuNPs. The characterization of Chi/AuNPs was carried out by transmission electron microscopy (TEM), scanning electron microscopy (SEM), Fourier-transform infrared (FTIR), and X-ray diffraction (XRD). Chi/AuNPs appeared spherical and monodispersed, with a diameter ranging between 20 to 120 nm. The synergistic effects of AuNPs and Chi led to the disruption of bacterial membranes. The maximum inhibitory impact was seen against *P. aeruginosa* at 500 µg/mL, with a zone of inhibition diameter of 26 ± 1.8 mm, whereas the least inhibitory effect was reported for *S. aureus*, with a zone of inhibition diameter of 16 ± 2.1 mm at the highest dose tested. Moreover, Chi/AuNPs exhibited antifungal activity toward *Candida albicans* when the MIC was 62.5 µg/mL. Cell viability and proliferation of the developed nanocomposite were evaluated using a sulphorhodamine B (SRB) assay with a half inhibitory concentration (IC_50_) of 111.1 µg/mL. Moreover, the in vitro wound-healing model revealed that the Chi/AuNP dressing provides a relatively rapid and efficacious wound-healing ability, making the obtained nanocomposite a promising candidate for the development of improved bandage materials.

## 1. Introduction

The emergence of multidrug-resistant (MDR) bacteria has become a severe threat to public health [1]. MDR bacteria are no longer isolated in medical settings; they may now be found in the environment around us. These bacteria include *Staphylococcus aureus, Enterococcus faecalis, Streptococcus pneumonia, Escherichia coli,* and *Klebsiella pneumoni* [2]. Gram-negative bacteria use different mechanisms to resist the toxicity of antibiotics, such as low permeability of the outer membrane, efflux pumps, and the production of degrading enzymes [3]. The true cost of antimicrobial resistance will be 300 million premature deaths and up to USD100 trillion (GBP64 trillion) lost to the global economy by 2050 [4]. On the other hand, fungal infections have significantly increased in the last decade in immunodeficient patients [5]. Globally, pathogenic fungi have invaded more than 1.2 billion individuals, causing at least 1.7 million deaths per year [6]. The recent annual incidences of invasive candidiasis, aspergillosis, and mucormycosis are over 750,000, 300,000, and 10,000 cases, respectively [7]. The widespread use of antifungal drugs leads to fungal strains resistant to most commercial antifungal agents [8]. Therefore, the discovery or synthesis of new antimicrobial agents is required.

Nanotechnology is assumed to be the subsequent industrial revolution and is considered to have a tremendous effect on the community, economics, and the common world [9,10]. An environmentally friendly approach to the biosynthesis of nanoparticles is an opportunity to be applied safely in medical fields [11,12]. Nanotechnology has received much attention in different biological applications [13]. Nanoparticles have been successfully used to reduce bacterial and fungal infections in medicine, pharmaceuticals, and agriculture fields [14,15,16,17]. Nanomaterials such as gold, silver, copper, selenium, titanium, zinc oxide, and magnesium oxide have antimicrobial activity against human pathogenic bacteria and fungi [18,19,20,21]. Among them, gold nanoparticles are considered one of the most commonly used metals for biomedical applications due to their unique properties, such as adjustable size, shape, surface properties, optical properties, biocompatibility, low cytotoxicity, and high stability [22,23,24]. In the majority of nanomaterials described in recent studies, antibacterial activity is attributed to at least one of the following mechanisms: inhibition of cell wall/membrane synthesis, disruption of energy conversion, production of toxic reactive oxygen species (ROS), photocatalysis, enzyme inhibition, and reduction of DNA production [25,26]. Polysaccharides such as cellulose, chitosan, and starch were added to nanoparticles to reduce aggregation and improve stability [27,28]. Chitosan is a linear polysaccharide that is obtained from the deacetylation of chitin, a naturally occurring polymer present in the shells of prawns and other crustaceans [29]. It is one of the most commonly used biopolymers in a wide range of applications, including fabrics, cosmetics, water treatment, and food processing [30,31,32]. Previous studies confirmed that chitosan has multiple roles in nanoparticle synthesis, stabilization, and applications [33,34]. Wound healing is characterized by a variety of reactions that include inflammatory, tissue-regenerating, and tissue-remodeling processes [35]. Wound dressings are biomaterials of synthetic or natural origin that aid in wound healing by establishing a favorable microenvironment, which attracts cells to the wound region [36]. In this work, an accurately designed synthesis of chitosan-based hybrid AuNPs based on this green and simple synthetic strategy, followed by accurate physicochemical characterization, will be described. After their physicochemical characterization, the wound-healing, antibacterial, and antifungal activities of the Chi/AuNPs will be investigated.

## 2. Materials and Methods

### 2.1. Materials

Chitosan (CS) was purchased from Sigma-Aldrich (Darmstadt, Germany). The molecular weight of CS is in the order of 45 kDa, and its degree of acetylation is about 80%, according to the manufacturer’s data. Glacial acetic acid (Panreac) and sodium hydroxide were obtained from Merck (Darmstadt, Germany). HAuCl4.3H2O (99.5%), Muller Hinton broth, resazurin indicator, nystatin, sulphorhodamine B, doxorubicin, TCA, TRIS, and ciprofloxacin were obtained from Sigma–Aldrich. All chemicals were of analytical grade and were used without further purification.

### 2.2. Synthesis of Chitosan/Gold Nanoparticles (Chi/AuNPs)

Chitosan/gold nanoparticles (Chi/AuNPs) were synthesized utilizing a chemical reduction process, with chitosan as a reducing and stabilizing agent. With minor changes, the synthesis of Chi/AuNPs was carried out according to the technique reported by [37]. Chitosan (0.2%) was produced by mixing in 0.5% acetic acid. After that, the chitosan solution was stirred to create a homogeneous solution. An aliquot of 2 mL of 2 mM HAuCl_4_.3H_2_O was added drop-by-drop to the chitosan solution. At 85 °C, the mixture was agitated for 4 h. The colorless chitosan solution became violet, indicating that Chi/AuNPs were synthesized.

### 2.3. Characterization of Chi/AuNPs

A variety of instrumental analytical methods were used to characterize the Chi/AuNPs. The shape and size of the prepared Chi/AuNPs were observed using the TEM method. An ultra-high-resolution transmission electron microscope (JEOL-2010, Jeol Ltd., Tokyo, Japan) with a voltage of 200 kV was employed. Specimens for TEM measurements were prepared using the drop coating method by placing a drop of diluted colloidal solution containing Chi/AuNPs on a copper grid coated by an amorphous carbon film and desiccating the solvent under vacuum overnight before loading onto a specimen holder. AMT software was calibrated for Chi/AuNP size measurements using a digital TEM camera. The average diameter of the prepared Chi/AuNPs was calculated by measuring over 100 nanoparticles in at least 10 random locations on the TEM grid in enlarged microphotographs. The electron diffraction of the selected area (SAED) was performed with TEM (JEOL-2010). SAED rings were measured, and the corresponding crystalline spaces were calculated with the relation d_hkl_ = K/D, where K is the camera constant of the microscope and D is the ring diameter. The interplanar distances d_hkl_ obtained were compared with the crystallographic data of NPs. A scanning electron microscope was used to study the topography of Chi/AuNPs; it has a resolution of ∼1.2 nm @ 30 kV. An electron probe was used to scan over the surface of the Chi/AuNPs, and these electrons interacted with the Chi/AuNPs. Secondary electrons were emitted from the surface of the Chi/AuNPs and recorded. The height differences in the Chi/AuNPs gave contrast to the image. A field emission scanning electron microscope, installed with a field emission gun (Quanta, 250-FEG, FEI, Hillsboro, OR, USA) and connected with an energy dispersive X-ray analyzer (EDX, Unit) with an excitation source of 30 kV for EDX and mapping, was used to examine the surfaces of the prepared Chi/AuNPs. Total internal reflectance/Fourier-transform infrared (ATR-FTIR) spectra were used to semi-quantitatively measure the observable IR spectrum of the Chi/AuNPs by evaluating the transmittance over a spectral region of 4000 to 400 cm^−1^, using a Spectrum Two IR spectrometer (PerkinElmer Inc., Shelton, CT, USA). To achieve a suitable signal quality, all spectra were collected at a 4 cm^−1^ resolution by collecting 32 scans. The XRD pattern of the Chi/AuNPs was carried out on a Diano X-ray diffractometer using a radiation source energized at 45 kV and a Philips X-ray diffractometer (PW 1930 generator, PW 1820 goniometer, where the resolution of the goniometer at *θ* and 2*θ* is 0.0001°) with a Cu K radiation source (*λ* = 0.15418 nm). The zeta potential and particle distribution of Chi/AuNPs in aqueous media were investigated using dynamic light scattering (DLS, Malvern Instruments Zetasizer Nano-ZS equipment, Malvern, UK). The distribution of the diffusion coefficients D of the particles was determined using decay times, which was then transformed into a distribution of hydrodynamic diameters 2R_H_ using the Stokes–Einstein formula R_H_ = k_B_T/6πηD, where k_B_T is the thermal energy and η is the solvent viscosity.

### 2.4. Cytotoxicity Assessment by Sulphorhodamine B (SRB) Assay

The normal human skin cell line BJ-1, obtained from the American Type Culture Collection (ATCC, Manassas, VA, USA), was used for investigating the cytotoxicity of the tested compounds by SRB assay [38]. Aliquots of 100 μL cell suspension (5 × 10^3^ cells) were put in 96-well plates and incubated in RMPI 1640 for 24 h. Before addition to the culture medium, tested Chi/AuNPs and standard drug doxorubicin (DOX) were dissolved in dimethyl sulfoxide (DMSO) and followed by serial dilution for 6 points, ranging from 200 to 1.56 μg/mL. After 72 h of exposure, cells were fixed by replacing media with 150 μL of 10% trichloro acetic acid (TCA) and incubated at 4 °C for 1 h. Aliquots of 70 μL SRB solution (0.4 % *w*/*v*) were added and incubated in a dark place at room temperature for 10 min. Plates were washed 3 times with 1% acetic acid and allowed to air-dry overnight. Then, 150 μL of unbuffered Tris base solution (TRIS) (Sigma-Aldrich) (10 mM) was added to dissolve the protein-bound SRB stain [39]. The test was conducted in triplicate. Results were recorded using a 450 nm absorbance value by Infinite-M200 Pro-TECAN (Tecan, Grödig, Austria). The percentage cell viability was calculated according to this equation:

(1) $$\mathrm{CT}\;\%=\frac{\mathrm{Ac}-\mathrm{At}}{\mathrm{Ac}\;}\times 100\;\%$$

where Ac and At are the absorbance of the control sample and the test sample, respectively.

Calculation of the half-maximal inhibitory concentration (IC50) is a suitable method for comparison of the activity of pharmaceutical materials. In this method, the measurement and comparison criterion is the concentration in which 50% of the final activity of Chi/AuNPs and standard drug doxorubicin (DOX). The graph of the IC50 of the Chi/AuNPs and standard drug doxorubicin (DOX) was produced by drawing the percent inhibition curve versus the tested compounds with different concentrations. The effective safe concentration at 100% cell viability (EC100) value of each tested extract was estimated by GraphPad Instat software (version 6.01, GraphPad, San Diego, CA, USA). Cytotoxic effects were categorized as cytotoxic (IC50 < 2.00 µg/mL), moderately cytotoxic (2.00 μg/mL < IC50 < 89.00 μg/mL), and non-toxic (IC50 > 90.00 µg/mL) according to the Special Programme for Research and Training in Tropical Diseases (WHO—Tropical Diseases). 

### 2.5. Cell Scratch Wound-Healing Assay

An in vitro wound-healing experiment was used to examine the wound-healing capacity of the final formula [40]. To achieve this, the human skin fibroblast cell line was seeded at a density of 3 × 10^5^/well onto a coated 6-well plate in 5% FBS-DMEM at 37 °C and 5 % CO_2_ [41]. The plate was then completely cleaned with PBS, the control wells were replenished with fresh medium, and the drug wells were treated with fresh media containing the drug. At the predetermined intervals, images were captured using an inverted microscope, and the plate was incubated at 37 °C with 5% CO_2_. The migration rate is defined as the proportion of wound closure area reduction, which increases as cells migrate over time

(2) $$\mathrm{Wound}\;\mathrm{closure}\;\%=\frac{A0-\mathrm{At}}{A0}\times 100\;\%$$

where A0 = 0 hr is the average wound area measured immediately after scratching (time zero), and At = h is the average wound area measured hours later.

### 2.6. Microbial Strains

*Staphylococcus aureus* ATCC^®^ 25923™, *Bacillus subtilis* ATCC 6633, extended-spectrum beta-lactamase (ESBL) *Klebsiella oxytoca* ATCC 51983, and *Pseudomonas aeruginosa* MTCC1034 were cultivated in Luria broth medium and incubated at 37 °C for 16–18 h. The fungal strains used were unicellular fungi (*Candida albicans* ATCC 90028) and multicellular fungi (*Aspergillus niger* RCMB 02724, *A. terreus* RCMB 02574, and *A. fumigatus* RCMB 02568). These four fungal strains were inoculated on MEA plates and incubated for 3–5 days at 28 ± 2 °C and then kept at 4 °C for further use [42,43,44,45].

### 2.7. In Vitro Susceptibility Testing

The antibacterial potential of synthesized Chi/AuNPs was determined by agar well diffusion assay against the tested selected bacterial strains [46]. All bacteria examined had a density of 0.5 McFarland at turbidity. Chi/Au-NPs were added to the bacterial culture in each well at a final concentration of 500 µg/mL and poured into each well separately, followed by a 24 h incubation at 37 °C. The size of the suppressive zone was assessed after incubation.

### 2.8. Antibacterial and Antifungal Activity of Chi/Au-NPs and Time-Kill Kinetic Assay

The minimum inhibitory concentration (MIC) of Chi/AuNPs was detected using the broth microdilution method of Chakansin et al., with minor modifications [47]. Chi/AuNPs (0–1000 µg/mL) were serially diluted twice in Muller Hinton broth with bacterial suspension (turbidity set to 5 × 10^5^ CFU/mL). The plate was incubated for 24 h at 37 °C. Then, 5 µL of resazurin indicator (made by dissolving 0.016 g in 100 mL of sterile distilled water) was added to each of the 96 wells. The micro-titer plate was then incubated in the dark. The color shift was then visually examined. Any changes in color from purple to pink or colorlessness were considered favorable, providing a direct indication of bacterial metabolic activity. The MIC value was determined as the lowest concentration at which the color change occurred. The MIC for the test material and the bacterial strain was computed as the average of three results. The time-kill dynamic test was performed in Mueller Hinton broth (MHB) using the methodologies provided by Hayat, et al. [48]. Chi/AuNPs were suspended in 1 mL of MHB medium (turbidity adjusted to 5 × 10^5^ CFU/mL), providing final concentrations of 0 MIC, 1 MIC, 2 MIC, 4 MIC, and 8 MIC for each type of bacteria in the final total volume of 1 mL. The cultures were incubated at 37 °C for 0, 1, 2, 3, and 4 h, with agitation at 100 rpm; 100 μL of culture was poured from the tubes onto Mueller Hinton agar (MHA) plates and incubated at 37 °C for 24 h. Each experiment was carried out in triplicate.

Antifungal activity of Chi/AuNPs was performed toward *C. albicans*, *A. terreus, A. niger,* and *A. fumigatus* using the agar well diffusion assay method [49], with minor modifications. Malt extract agar (MEA) plates were used for growing tested fungal strains at 30 °C for 3–5 days [50,51,52]. One mL of fungal suspension (10^7^ spores/ mL) was put and distributed on MEA plates. Agar wells (7 mm) were separately filled with 100 µL of Chi/AuNPs, Au, Chi, and nystatin and then incubated at 30 °C. Afterward, the inhibition zone diameter was measured [53,54]. The MIC of all test materials toward all tested fungal strains was assessed using the broth microdilution technique according to the standard European Committee on Antimicrobial Susceptibility Testing (EUCAST) methodology [55].

### 2.9. Statistical Analysis

Data are presented as means ± standard deviation (SD) of at least three independent experiments. Comparisons of data were made by Student’s *t*-test or by ANOVA when appropriate. Differences were considered statistically significant at *p* < 0.05. Statistical analysis was carried out and estimated using GraphPad Instat software.

## 3. Results and Discussion

### 3.1. Characterization of Chi/AuNPs

**Figure 1:**
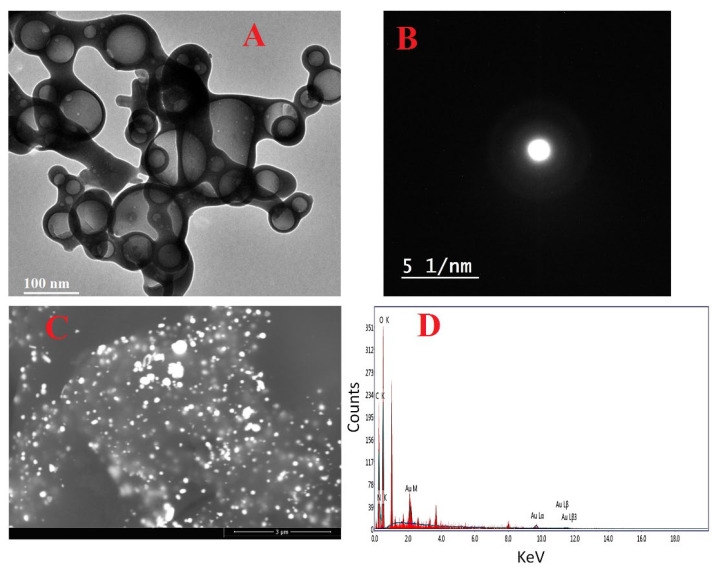
TEM images (**A**), SAED pattern (**B**), SEM image (**C**), and (**D**) EDX spectrum of Chi/AuNPs.

Nanostructures have piqued curiosity as a fast-evolving class of materials with a wide range of uses. Whenever a synthesis process is carried out, it is important to determine either the structure or composition of the end product, which may be accomplished by utilizing a variety of approaches, ranging from structural elucidation to determining the purity of the product under investigation [56]. In the present study, the Chi/AuNP nanocomposite was successfully synthesized. The most effective technique for determining the morphological structure and size of a prepared nanostructure is TEM. The TEM image indicated that the generated Chi/AuNPs were spherical and had sizes in the range of 25–100 nm (Figure 1A). The particles appeared spherical, with a thin layer of chitosan around the gold core. In addition, TEM micrographs showed uniform layers of chitosan covering the gold nanostructures. The gold particles appeared to be coated with a layer of chitosan, validating the generation of Chi/AuNPs. The area-selected electron diffraction (SAED) pattern of Chi/AuNPs is shown in Figure 1B, which demonstrates good sharp-rings and confirms the Au-nanostructures’ crystalline structure [57,58]. As shown in Figure 1C, SEM was used to evaluate the surface morphology and particle size of Chi/AuNPs. The particle size varied from 20 to 120 nm on average. EDX analysis was used to determine the elemental composition of the Chi/AuNP powder. In the Chi/AuNPs, the EDX spectra revealed the existence of several well-defined bands associated with the gold (Au), oxygen (O), and carbon (C) components (Figure 1D). C and O signals come from the chitosan, whereas the gold (Au) peak indicates the formation of Au-nanostructures. Furthermore, EDX spectra revealed the generation of very pure Chi/AuNPs with no additional impurity-related peaks. The morphological structure of Chi/AuNPs matched that of the chitosan-reduced gold particles previously reported [59].

**Figure 2:**
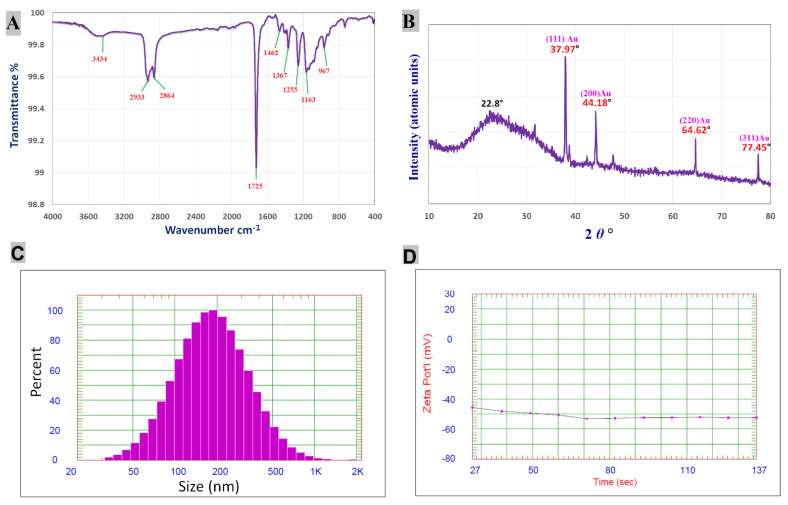
FTIR spectrum (**A**), XRD pattern (**B**), particle size distribution (**C**), and zeta potential (**D**) of Chi/AuNPs.

FT-IR analysis was conducted to detect the functional groups responsible for reduction, capping, and stabilizing synthesized Chi/AuNPs. The FTIR spectra of AuNPs revealed absorption peaks at 3434, 2933, 2864, 1725, 1462, 1367, 1255, 1163, and 967 cm^−1^, which correspond to linkage groups (Figure 2A). Moreover, the peaks at 3434 cm^−1^ matched the OH group stretching vibrations. The peaks at 2933 and 2864 cm^−1^ are attributed to the stretching of C−H groups. Carbonyls expand vibrations in aldehydes, ketones, and carboxylic acids, which correlate to the peak at 1725 cm^−1^. The existence of a strong 1725 cm^−1^ band in Chi/AuNPs shows that gold ion (Au^+^) reduction is accompanied by hydroxyl group oxidation in chitosan structures. C-N- and -NH stretching are shown by the peaks seen at 1462 and 1367 cm^−1^ [60]. The peak at 1255 cm^−1^ can be attributed to -C–O–C stretching. C–O stretching vibration is shown by the peak at 1163 cm^−1^. The absorption peak at 967 cm^−1^ conforms to the β-D glucose unit’s typical absorption.

One of the most extensively used methods for characterizing NPs is X-ray diffraction (XRD). The crystalline nature, phase behavior, and lattice constants are commonly determined using XRD. The XRD pattern (Figure 2B) of Chi/AuNPs shows characteristic diffraction peaks at 37.97°, 44.18°, 64.62°, and 77.45°, corresponding to (111), (200), (220), and (311). Bragg’s reflection is in good agreement with the face-centered cubic (FCC) structure of AuNPs (JCPDS card no: 04-0784). Furthermore, the most prominent diffraction peak at 22.8° confirmed the crystalline form of chitosan [37]. The current XRD results of Chi/AuNPs were consistent with previous findings of chitosan-mediated AuNPs [57]. DLS is one of the most common techniques used for detecting the intensity weight distribution of particle sizes in a colloid solution. The obtained Chi/AuNPs were a poly-dispersed mixture with an average diameter of 218.2 nm (Figure 2C). The size of the Chi/AuNPs was found to be higher in the DLS results compared to TEM analysis due to water molecules around the Chi/AuNPs [21,61]. The zeta potential measurement of the particle surface charge was used to assess the composite’s stability. There is agreement from previous studies that nanoparticles are affected by zeta values in solutions, which stabilize nanoparticles whether the zeta values are negative or positive [21,62,63]. The synthesized Chi/AuNPs were highly stabled as they have a zeta potential of −52.39 mV (Figure 2D). This high value confirms the high stability of the colloidal solution.

### 3.2. Cytotoxicity on Normal Human Skin Cell Line (BJ-1) 

**Figure 3:**
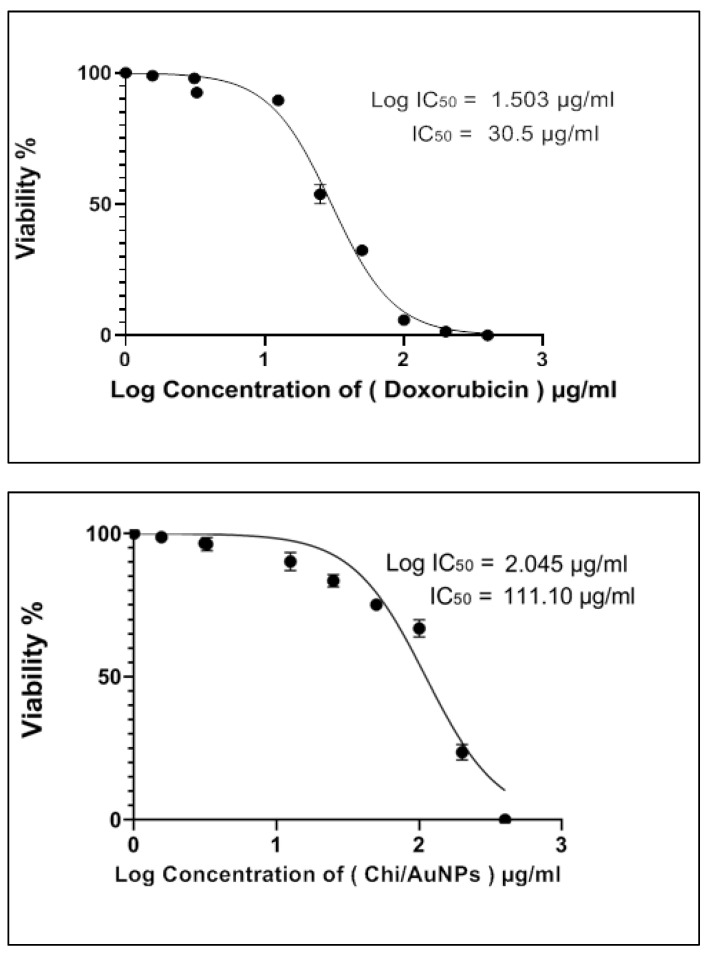
In vitro cytotoxicity effects on doxorubicin and Chi/AuNPs against the normal human skin cell line (BJ-1), assessed by SRB colorimetric assay, respectively.

To determine the sensitivity of the normal human skin cell line BJ-1 to the cytotoxic effects of Chi/AuNPs, the cells were seeded into microplates and incubated with various concentrations of Chi/AuNPs, as mentioned under the section on materials and methods. The results revealed that the Chi/AuNPs produced were non-toxic to normal skin cells; the prevention of unwanted dissolution of AuNPs would give less cytotoxicity or better biocompatibility. Doxorubicin and Chi/AuNPs have half-maximal inhibitory concentrations (IC_50_s) of 30.5 and 111.1 µg/mL, respectively (Figure 3). Furthermore, it was shown that doxorubicin was more harmful to the normal human skin cell line than Chi/AuNPs. As a result, Chi/AuNPs were determined to be non-toxic (IC_50_ > 90.00 µg/mL) by the Special Programme for Research and Training in Tropical Diseases (WHO—Tropical Diseases). Nanoparticles may cause toxicity after entering the body due to their unique physicochemical properties, including a large surface area that can enhance their biological effect [64]. As a result, given that the specific mechanisms and pathways through which nanoparticles may exert their toxic effects are largely unknown, evaluation of the potential toxic effects of these nanoparticles in the body is necessary [64].

### 3.3. Cell Migration Assay (Wound Scratch Assay)

**Figure 4:**
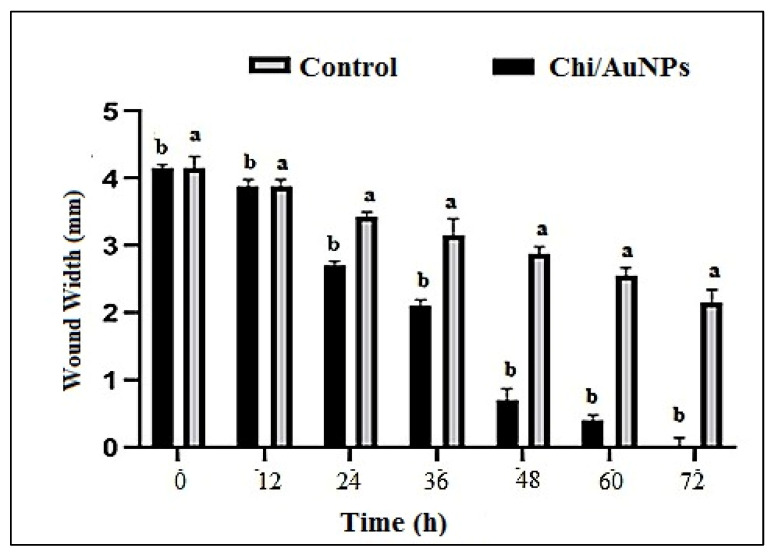
Effects of different treatments on wound area contraction (0–72 h). Values are given as mean ± SD (n = 3/group). Different letters indicate significant differences (*p* < 0.05).

**Figure 5:**
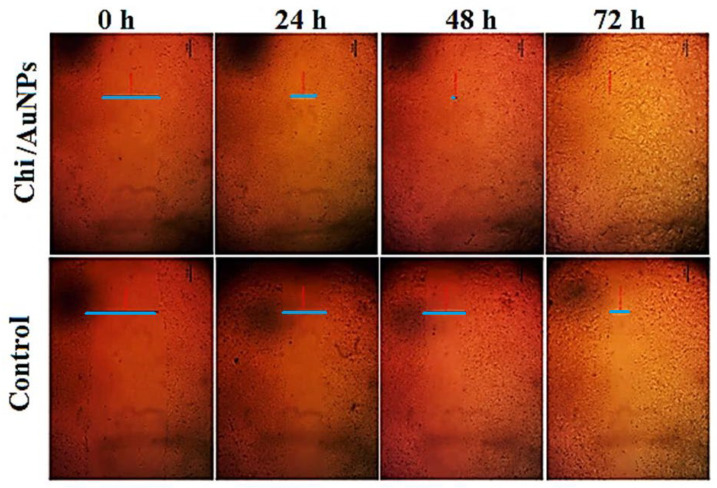
Representative micrograph pictures of cells treated with 100 μg/mL of the tested compound (Chi/AuNPs) and untreated (control) at 0 and 24 h. Wound closure rates are expressed as a percentage of scratch closure after 0 to 96 h compared to the initial area.

For its unique role, including proliferation and differentiation in healing wounds, human skin fibroblast cells have been frequently employed to examine epidermal abnormalities. In vitro wound-healing studies have been carried out on human skin fibroblasts, and the corresponding results are presented in Figure 4 and Figure 5. Wounds treated with 100 µg/mL Chi/AuNPs significantly (*p* < 0.001) reduced the wound area at 0, 12, 24, 36, 48 and 72 postoperative hours (Figure 4). In this study, no significant difference in wound contraction was observed in the experimental group after 12 h of treatment. However, a significant difference in wound contraction rate was observed on 24, 36, 48, and 72 h between the control and experimental group (*p* = 0.029). As shown in Figure 5, the results showed that the average percentage of wound contraction was significantly increased in the treated group 93.1 ± 0.71 %, 65.8 ± 0.91%, 46.3 ± 0.61%, 17.3 ± 0.61 %, 7.5 ± 0.51%, and 2.1 ± 0.31% contraction at 12, 24, 36, 48, 60, and 72 postoperative hours. The percentage of wound contraction in the control group was found to be 95.1 ± 1.41% at 12 h, 82.9 ± 4.07% at 24 h, 75.5 ± 2.12% at 36 h, 70.7 ± 1.22% at 48 h, and 51.2 ± 3.12% at 72 h. A significant difference ((A) *p* < 0.001 and (B) *p* < 0.05) can be observed between the control and Chi/AuNPs.

In a related study, nanocomposites based on chitosan demonstrated their capacity in the proliferative phase of the wound-healing process; due to their anti-inflammatory impact, biocompatibility, retention of fibroblast growth factors, and stimulation of human skin fibroblast activities, chitosan has been widely employed as a wound dressing material [62]. In another application of wound healing, nanomaterials were successfully used to generate nanopolymeric scaffolds that mimic properties such as Chi/AuNPs, which have been widely explored for their possible antibacterial properties. As an alternative to standard oral and parenteral routes, many types of nanocarriers have been used to increase medication absorption through the skin. Chi/AuNPs caused less postoperative infection and faster recovery than the control group. To aid wound healing, AuNPs may be readily integrated and cross-linked with collagen, gelatin, and chitosan [63]. This functionalization approach aids the biocompatibility and biodegradability of AuNPs.

### 3.4. In Vitro Susceptibility Testing and Time-Kill Kinetic Assay

Gold nanoparticles in different dimensions and shapes are the most widely studied nanomaterials for antibacterial applications [65]. The preliminary detection of Chi/AuNPs against tested pathogenic bacteria Gram-negative bacteria extended-spectrum beta-lactamase (ESBL) *Klebsiella oxytoca* ATCC 51983 and *Pseudomonas aeruginosa* MTCC1034 and Gram-positive bacteria *Staphylococcus aureus* ATCC^®^ 25923™ and *Bacillus subtilis* ATCC 6633 was done using the agar well diffusion method and broth microdilution assay. The diameter of the zone of inhibition ranged from 14 to 26 mm. The maximum inhibitory impact was seen against *P. aeruginosa*, with a zone of inhibition diameter of 26 ± 1.8 mm, whereas the least inhibitory effect was detected for *S. aureus,* with a zone of inhibition diameter of 16 ± 2.1 mm. Due to the presence of a thick layer of peptidoglycan in the cell wall, Gram-negative bacteria such as *P. aeruginosa* and *E. coli* are more sensitive to biogenic Chi/AuNPs than Gram-positive bacteria, according to Baskaran et al. [66]. The antibacterial action could be attributed to the synergistic effect of Au-NPs with chitosan [67]. Agar well diffusion methods have been used as preliminary tests to investigate the antimicrobial activities of a variety of medicinal drugs; MIC determination was used to further evaluate the antibacterial activities of Chi/AuNPs [68]. The MIC is the lowest concentration of Chi/AuNPs necessary to prevent observable microbial growth [68]. The lowest concentration at which color change occurred was taken as the MIC value [69,70]. Our results showed that Chi/AuNPs possess MIC values ranging from 1.56 to 6.25 μg/mL (Table 1). Some investigations have shown that AuNPs can enter the cell wall, causing damage to cell membrane permeability, disrupting cell respiration processes, stimulating the generation of free radicals, and inactivating cellular proteins by gold ions [69,71].

**Figure 6:**
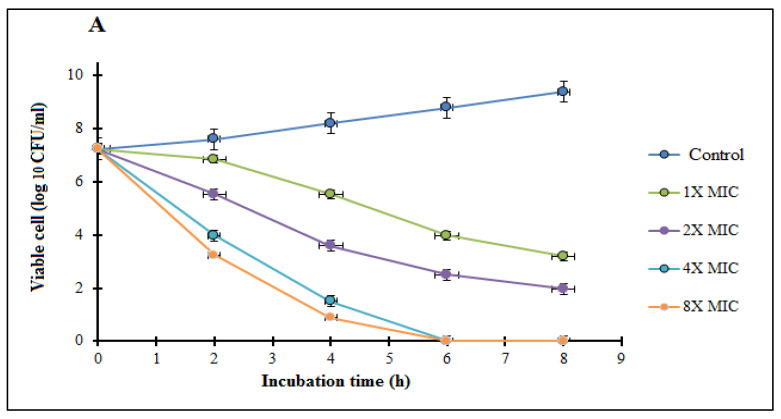

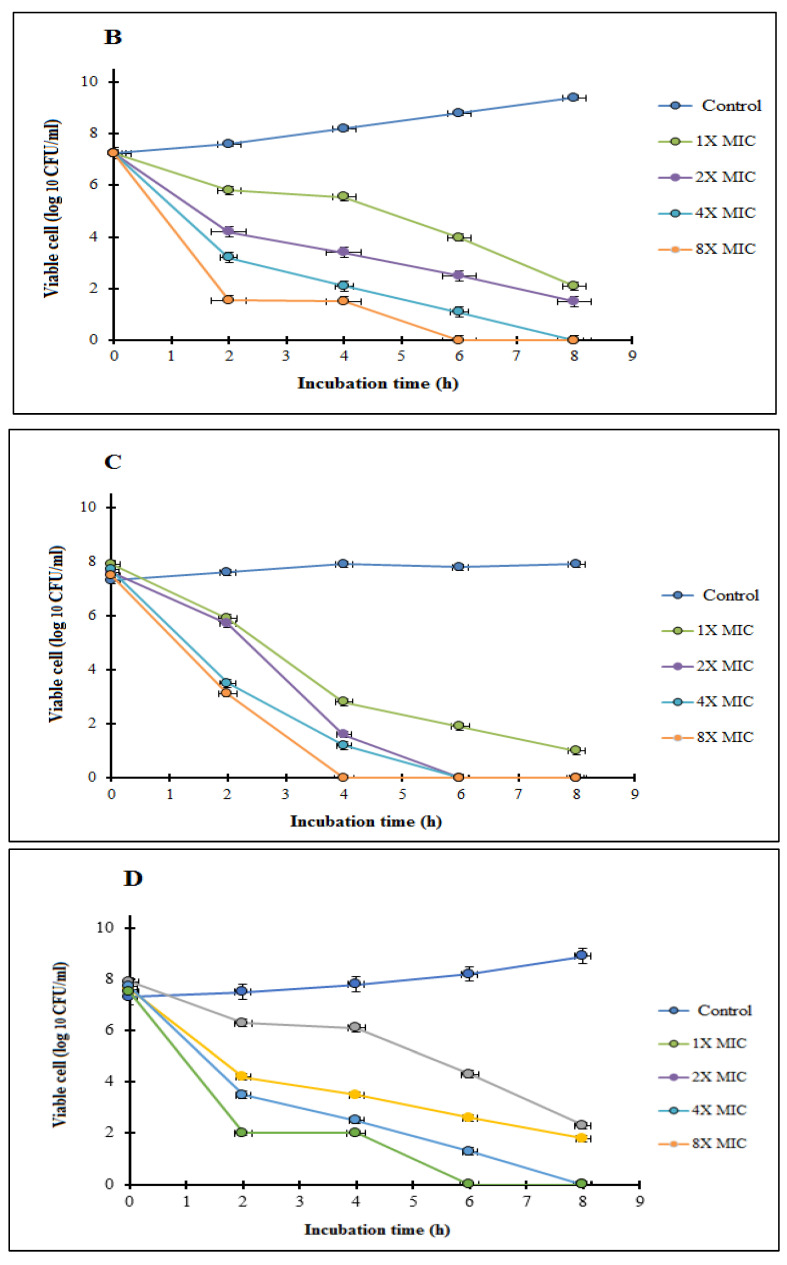
Time-kill plots of Chi/AuNPs against human pathogenic bacterial strains A: *Staphylococcus aureus* (**A**), *Pseudomonas aeruginosa* (**B**), *Bacillus subtilis* (**C**), and *Klebsiella oxytoca* (**D**) at different concentrations and time length. The experiment was performed in triplicate and a graph of the log CFU/mL was plotted against time.

One of the most essential elements of bacterial pathogenicity is their high reproduction rate, which can be efficiently addressed to minimize viable bacterial infections [72]. The time-kill kinetics of the tested compound against selected bacterial strains at the test concentrations studied (0, 1, 2, 4, 8 MIC) of Chi/AuNPs are shown in Figure 6. These findings validate the bactericidal ability of Chi/AuNPs. The time-kill kinetics profile of the biosynthesized Chi/AuNPs demonstrates a progressive decrease in the number of viable cells (CFU/mL) over time. The number of selected Gram-negative bacteria was reduced at 4 and 6 h. The bactericidal endpoint of Chi/AuNPs for *K. oxytoca* and *P. aeruginosa* were reached after 4 h of incubation with 8× MIC (24.96 and 12.48 µg/mL, respectively). After 6 h, bactericidal activity was investigated at 4× MIC (12.48 and 6.24 g/mL, respectively) and 2× MIC (6.24 and 3.12 g/mL, respectively). The time-kill kinetics profile of the Chi/AuNPs against the tested Gram-positive bacteria was reached after 6 h of incubation at 8× MIC (50 µg/mL) and at 4× MIC (25 µg/mL) after 8 h. Gold nanoparticles with antibacterial properties may have a twofold mode of action when compared with chitosan only. Chitosan is a well-known stabilizer for metal nanoparticles in biomedical engineering. The inhibitory mechanism is based on the interaction of the positive charge Chi with negatively charged biomolecule residues on the bacterium cell surface under acidic conditions [21,73,74].

### 3.5. Antifungal Activity

**Figure 7:**
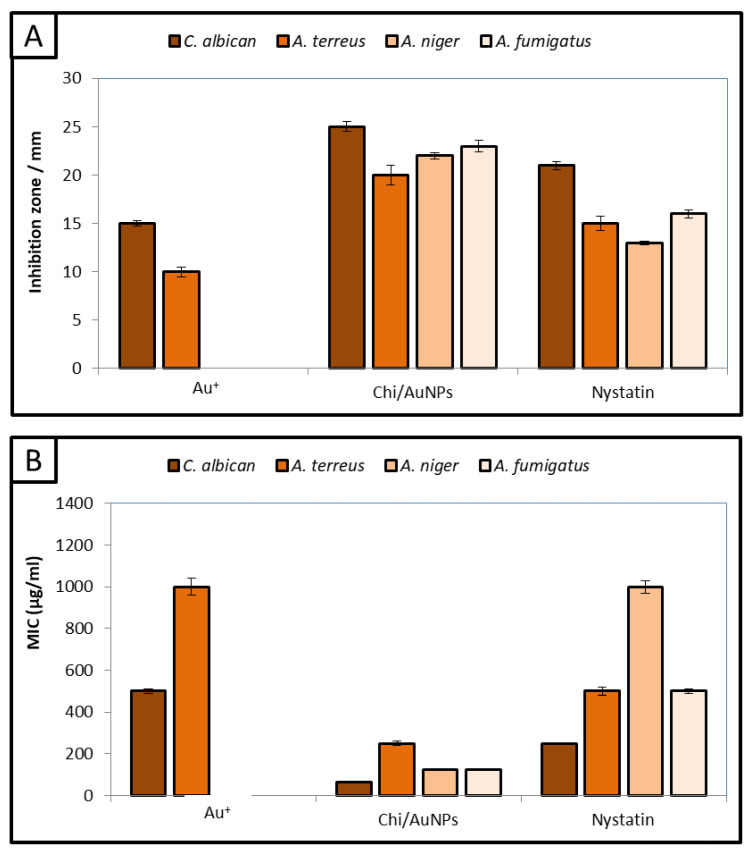
Antifungal activity (**A**) and minimum inhibitory concentration (**B**) of Chi/AuNPs, chitosan, HAuCl4.3H2O, and nystatin against *C. albicans*, *A. terreus*, *A. niger*, and *A. fumigatus*.

The antifungal activity of Chi/AuNPs against human pathogenic fungi has rarely been reported. Therefore, the antifungal activity of Chi/AuNPs was evaluated against *C. albicans, A. terreus, A. niger,* and *A. fumigatus*. Uni- and multicellular fungi were used for evaluating the antifungal activity of Chi/AuNPs, as shown in Figure 7A. The results illustrated that Chi/AuNPs have outstanding antifungal activity against tested uni- and multicellular fungi. Moreover, results showed that Chi/AuNPs have antifungal activity toward unicellular fungi more than multicellular fungi, where the inhibition zone of Chi/AuNPs (2000 µg/mL) against *C. albicans* was 25 mm compared to nystatin 21 mm. Furthermore, the inhibition zone of Chi/AuNPs against *A. terreus, A.niger,* and *A. fumigatus* was 20, 22, and 23 mm, respectively. On the other hand, Au^+^ has weak antifungal activity toward *C. albicans* and *A. terreus* only, while it does not have antifungal activity towards *A.niger and A. fumigatus.* Additionally, chitosan did not exhibit any antifungal activity against all tested fungal strains. There have been many studies on Au(I) and Au(III) reporting their antimicrobial activity against a wide variety of microorganisms [75,76]. Additionally, previous studies have reported that AuNPs have antifungal activity against *Candida* spp. [73,77]. Ahmad, et al. [74] reported that AuNPs have excellent size-dependent antifungal activity and biocidal action against *Candida* isolates. Likewise, Wani and Ahmad [78] confirmed that AuNPs have promising antifungal activity toward Candida species. Mondal, et al. [79] illustrated that AuNPs have potential antifungal activity against aspergillus species.

Furthermore, MICs of Chi/AuNPs and Au^+^, Chi, and nystatin were detected, as shown in Figure 7B. The results illustrated that MIC_s_ of Chi/AuNPs toward *C. albicans, A. terreus, A. niger,* and *A. fumigatus* were 62.5, 250, 125, and 125 µg/mL, respectively. According to the cytotoxicity test of Chi/AuNPs in this study (Figure 3), the IC_50_ of Chi/AuNPs was 111.1 µg/mL. Consequently, Chi/AuNPs can be used as an antifungal agent against *C. albicans* only where their MIC is lower than the IC_50_ of Chi/AuNPs. On the other hand, using Chi/AuNPs as antifungals against *A. terreus, A. niger,* and *A. fumigatus* is not recommended due to their toxicity in the normal cell line, where the MIC was greater than the IC_50_ of Chi/AuNPs. The antifungal activity of Chi/AuNPs is attributed to combining the positive charge of the amino group in chitosan with the negative-charge components of fungal cells. Therefore, Chi/AuNPs may inhibit fungal growth by chelating various transitional metal ions, inhibiting enzymes. AuNPs based on chitosan have been widely used for the inhibition of bacterial growth and biofilm [80,81,82]. Additionally, a succinyl–chitosan gold nanocomposite was used to inhibit the growth of *C. albicans* [83]. Moreover, AuNPs based on chitosan have been studied against *C. albicans, Fusarium solani,* and *A. niger,* where the MIC was greater than 250 µg/mL for all strains [84].

## 4. Conclusions

Over the last several decades, chitosan has attracted a lot of interest and attention due to its wide range of prospective applications and distinctive benefits. In this study, a nanocomposite based on AuNPs and chitosan was fabricated through a facile method. Chi/AuNPs appeared spherical and monodispersed, with a diameter range of 20 to 120 nm. This nanocomposite has promising antibacterial activity against Gram-negative and Gram-positive bacteria. Likewise, it has potential antifungal activity against unicellular and multicellular fungi. Furthermore, Chi/AuNPs are safe to use due to them not affecting the normal human skin cell line. Antimicrobial resistance may be improved by adding gold nanoparticles into chitosan hydrogels, which lowers bacterial infection and improves wound healing.

## Figures and Tables

**Table 1:** Inhibition zones and MICs of Chi/AuNPs, chitosan, HAuCl_4_.3H_2_O, and ciprofloxacin.

	*S. Aureus*		*B. subtilis*		*P. Aeruginosa*		*K. Oxytoca*	
	IZ/mm (500 µg/mL)	MIC (µg/mL)	IZ/mm (500 µg/mL)	MIC (µg/mL)	IZ/mm (500 µg/mL)	MIC (µg/mL)	IZ/mm (500 µg/mL)	MIC (µg/mL)
**Chi/AuNPs**	16 ± 2.1	6.25	19 ± 1.8	6.25	26 ± 1.8	1.56	22 ± 1.8	3.12
**Chitosan**	9.1 ± 1.9	50	8.7 ± 2.4	50	9.8 ± 3.9	50	8.2 ± 2.8	50
**HAuCl_4_.3H_2_O** (Au^+^)	ND	ND	ND	ND	ND	ND	ND	ND
**Ciprofloxacin**	14 ± 3.2	25	16 ± 1.9	50	22 ± 2.4	50	21 ± 2.9	50

MIC: minimum inhibitory concentration; IZ/mm: diameter of inhibition zone (mm).

## Data Availability

The data used to support the findings of this study are available from the corresponding author upon request.

## Abbreviations

Chi/AuNPs: gold nanoparticles loaded with chitosan; Chi: chitosan; AuNPs: gold nanoparticles; MDR: multidrug-resistant; TEM: transmission electron microscope; SEM: scanning electron microscope; BJ-1: human skin cell line; SRB: sulphorhodamine B; IC_50_: half-maximal inhibitory concentrations; MIC: minimum inhibitory concentration; MBC: minimum bactericidal concentration.
